# Vegetation History and Survival Patterns of the Earliest Village on the Qinghai–Tibetan Plateau

**DOI:** 10.3389/fpls.2022.903192

**Published:** 2022-05-12

**Authors:** Jingyi Gao, Guangliang Hou, Yongming Xiao, Chongyi E, Haicheng Wei, Yongjuan Sun, Manping Sun, Hongpan Xue, Zhuoma Wende, Sunmei Jin, Xiaoliang Chen

**Affiliations:** ^1^Key Laboratory of Tibetan Plateau Land Surface Processes and Ecological Conservation (Ministry of Education), College of Geographical Science, Qinghai Normal University, Xining, China; ^2^Qinghai Province Key Laboratory of Physical Geography and Environmental Process, College of Geographical Science, Qinghai Normal University, Xining, China; ^3^Academy of Plateau Science and Sustainability, People's Government of Qinghai Province and Beijing Normal University, Xining, China; ^4^Qinghai Provincial Institute of Cultural Relics and Archaeology, Xining, China; ^5^Key Laboratory of Comprehensive and Highly Efficient Utilization of Salt Lake Resources, Qinghai Institute of Salt Lakes, Chinese Academy of Sciences, Xining, China; ^6^School of Earth and Space Sciences, University of Science and Technology of China, Hefei, China

**Keywords:** Qinghai–Tibetan Plateau, Epipaleolithic to Bronze Age, end-member modeling analysis, pollen, vegetation change, survival patterns

## Abstract

The upper Yellow River valley in the northeastern Qinghai–Tibetan Plateau (QTP) is an important corridor for prehistoric migration to the hinterland plateau. However, most studies have focused on the Neolithic Age, with limited evidence for earlier periods. The Shalongka (SLK) site on the northeastern QTP spans the Epipaleolithic to Bronze Age and contains cultural deposits, so provides a good basis for unraveling the evolutionary history of the human-land relationship. In this study, we sampled the 420-cm-thick section T1406E at the SLK site and undertook lithologic stratigraphic description and analysis of grain size, redness, magnetic susceptibility, geochemical elements, pollen and charcoal. Dating control was provided by accelerated mass spectrometry ^14^C and optically stimulated luminescence methods. Results show that SLK site was affected by the local fluvial sedimentary environment. The absolute dating results of the SLK site have revealed that humans occupied the site during the Epipaleolithic (8.5–7.3 cal ka BP), Yangshao culture (5.9–5.1 ka) and Qijia Culture (4.1–3.9 cal ka BP). Pollen analysis showed that the humans lived in a landscape that was predominated by forest-steppe. Consolidating with multidisciplinary evidence, we learned that Epipaleolithic sites were occupied by microlithic hunter-gatherers and comprised by relatively fixed seasonal central campsites, and their mobility was significantly decreased from the early to late period. Subsequently, farmers of the Yangshao culture migrated from the low elevation (Chinese Loess Plateau) to the upper Yellow River valleys on the QTP and founded the earliest settlement villages (~5.9 ka) on the QTP. People of the Qijia culture adopted diversified survival strategies under the settled lifestyle. In all, we infered that SLK site may play an important role in the communication and integration between different people and cultures.

## Introduction

The location of the Qinghai–Tibetan Plateau (QTP), at the interface of the East Asian summer monsoon (EASM), Indian summer monsoon (ISM) and Westerlies system, makes it particularly sensitive to climate change (An et al., 2012; Li et al., 2018). Moreover, the QTP represents a great challenge to the survival and communication of prehistoric humans because of its high average elevation of >4,000 m above sea level (a.s.l.), hypoxic environment and sparse faunal and floral resources (Beall, 2001). The northeastern QTP is at the transition between the western Chinese Loess Plateau (CLP) and the eastern QTP, which comprises a typical agro-pastoral ecotone; it was an important area for the exchange of different types of prehistoric cultures due to its relatively lower elevation compared to other parts of the QTP (Zhang et al., 2016; Gao et al., 2020). Thus, studies of past human-environment interactions in the area help our understanding of the evolution, trajectory and mechanisms of prehistoric human adaptation to extreme environments (Dong, 2018).

In the past few decades, many studies has been carried out on the northeastern QTP to explore the history of expansion of prehistoric humans to the inner QTP, including the timing, location, route and subsistence strategies of migrants and their relationship with the environment (Madsen et al., 2006; Brantingham et al., 2007; Rhode et al., 2007; Sun et al., 2012; Dong et al., 2013; Chen et al., 2015, 2019; Hou et al., 2015; Zhang D. J. et al., 2020). For example, studies based on archaeology, genetics and chronology show that upper Yellow River and its tributary valleys in the northeastern QTP provide a crucial corridor for prehistoric human migration to the hinterland of the plateau (Madsen et al., 2006; Li et al., 2019). However, previous studies on upper Yellow River valleys mostly focused on comparative analysis of environmental changes and human activities on the large scale, so that detailed geomorphological investigation of archaeological sites is generally lacking. Also, the studies have concentrated on human–land relationships since the Neolithic, with little on the Paleolithic to Epipaleolithic to transition (Gao et al., 2008), due to limited archaeological sites dating to this period.

To date, only one site spanning the Epipaleolithic to the historical period with stratigraphic sedimentation has been identified in the upper Yellow valley on the northeastern QTP, at Shalongka (SLK) site (Chinese Society of Archaeology, 2017). There has been some work on the chronology (Dong et al., 2013; Wang Z. L. et al., 2021), zooarchaeology, archaeobotany, and paleoenvironment of the site (Li et al., 2014; Chen et al., 2015; Yi et al., 2020). The chronological framework and subsistence strategies at SLK site have been preliminarily explored, based on archaeological investigation and test excavation (Madsen et al., 2006; Rhode et al., 2007), but there has been no systematic archaeological excavation of the site. In addition, prehistoric hunter-gatherers were usually highly mobile and migrated regularly, leaving only patchy remnants of their activity, thus the evidence can only provide limited and discontinuous information on human activities (Ledger, 2018). In this context, the SLK site is critical; systematic archaeological excavation and multi-proxy analysis is needed to clarify the environmental background, occupation and adaptation history, the transition from Epipaleolithic to Neolithic, and relationship with the peoples of the low elevation northern China.

In this study, we investigated the east wall of section T1406 (T1406E) as part of the first formal archaeological excavation of the SLK site in Qunjian Basin. We established a chronological framework based on accelerated mass spectrometry radiocarbon (AMS^14^C) dating of charcoal from the section and optically stimulated luminescence dating (OSL) of pottery shards. Environmental samples were collected for multi-proxy analysis, including grain size (GS), magnetic susceptibility (MS), redness (a^*^), pollen, charcoal and geochemical elements. Combining our results with archaeological data, paleoclimatology, genetic and linguistic evidence, we aimed to: (1) reconstruct the environmental background (sedimentary environment and vegetation evolution) and history of prehistoric human activity at the SLK site; (2) explore the mobility pattern of prehistoric humans in different eras.

## Regional Setting and Study Site

**Figure 1:**
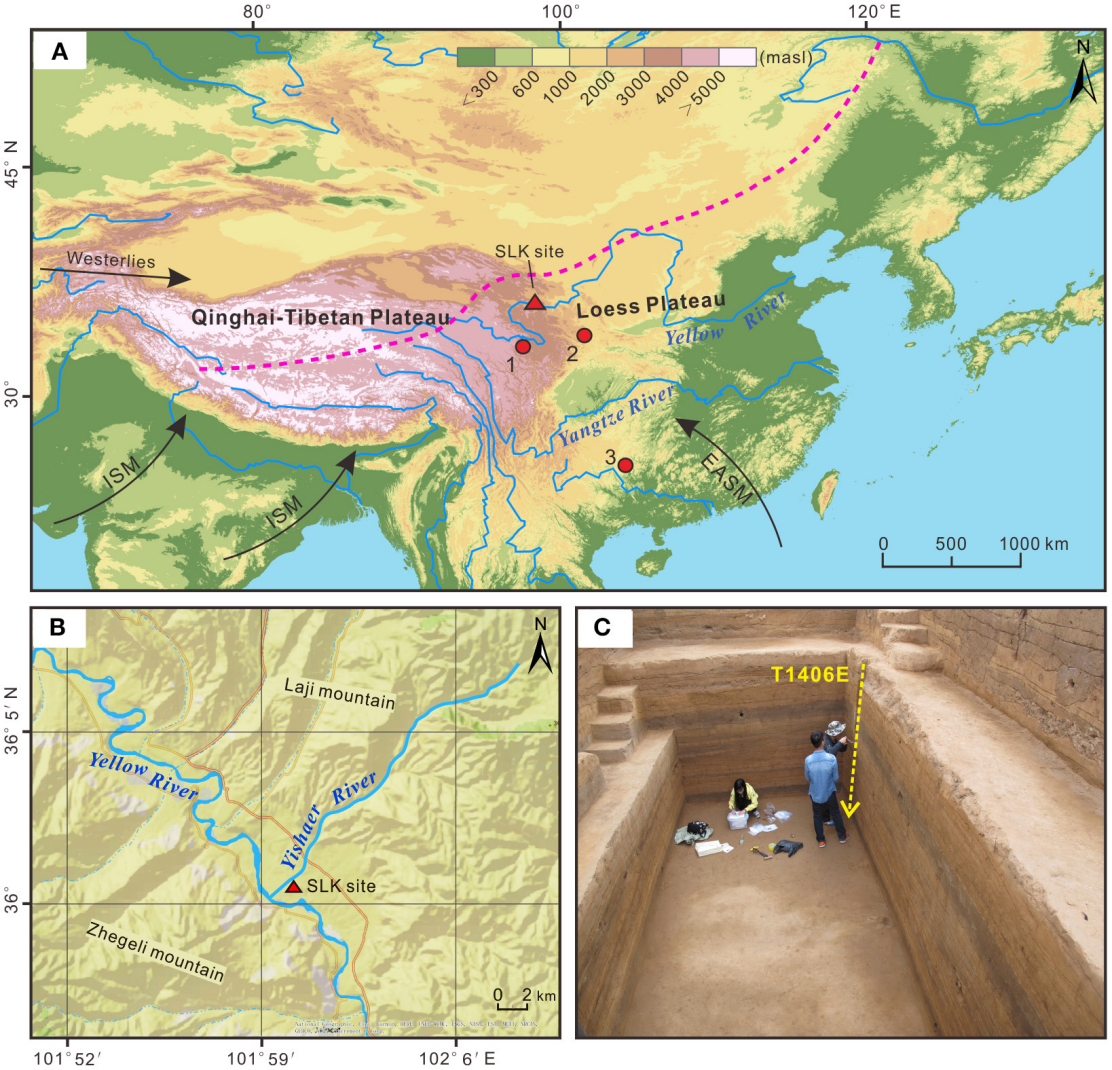
Overview of the study area. **(A)** Location of Shalongka (SLK, red triangle) site and nearby sites with climate records (red circles). 1. Peat section in the Zoige Basin (Liang et al., 2020); 2. Wuya Cave stalagmite in the western Chinese Loess Plateau (Tan et al., 2020); 3. Dongge Cave stalagmite, Guizhou Province, China (Dykoski et al., 2005). The dashed purple line indicates the modern northern limit of the Asian Summer Monsoon (Chen et al., 2008). EASM denotes East Asian summer monsoon, ISM denotes Indian Summer Monsoon. **(B)** Location of the SLK site in the Qunjian Basin. **(C)** Location of the sampling column in section T1406E (yellow dotted line) at SLK site.

Qunjian Basin is a small intermountain basin in the upper reaches of the Yellow River, in southwestern Hualong county, Qinghai Province (Figures 1A,B). The basin is ~24 km long, from the eastern entrance of Lijia Gorge in the west to the western entrance of Gongbo Gorge in the east, and 4 km wide, between Laji mountain to the north and Zhegeli mountain to the south, covering an area of 2,950 km^2^ (Figure 1B). Elevation gradually decreases from northwest to southeast, averaging ~2,050 m a.s.l. The Yellow River traverses the basin from west to east, making it to be an important focus for human activity (Wang Z. L. et al., 2021). At the present day, average annual temperature is 7.8°C, and average annual precipitation is 357.3 mm, of which 70% occurs in summer (Jia, 2012).

The SLK site (36.01°N, 102.00°E; 2021 m a.s.l.) is on the second terrace in Qunjian Basin, at the junction of the Yellow River and Yishaer River. The site covers an area of around 2.4 × 10^4^ m^2^; it is ~500 m from the Yellow River in the south, 24 m above the river, and ~260 m from the Yishaer River in the west, 10 m above the river (Figure 1B). SLK site was first discovered in 1987 and formally excavated by Qinghai Provincial Institute of Cultural Relics and Archaeology in 2003 and 2016. Cultural remains including house sites, post holes and hearths were found, along with a large numbers of cultural artifacts including lithics, pottery and spinning wheel (Chinese Society of Archaeology, 2017). Today, the area around the site is mainly used for agricultural cultivation, with orchards, corn and wheat. Overall, the SLK site has abundant prehistoric cultural remains that provide ideal material for investigating the long-term evolution of human-land relationships on the QTP. Based on Zhang (2000) proposal that “Epipaleolithic cultures are Paleolithics in the Holocene,” this study termed Paleolithic culture as “Epipaleolithic.”

## Materials and Methods

### Field Work and Sampling

**Figure 2:**
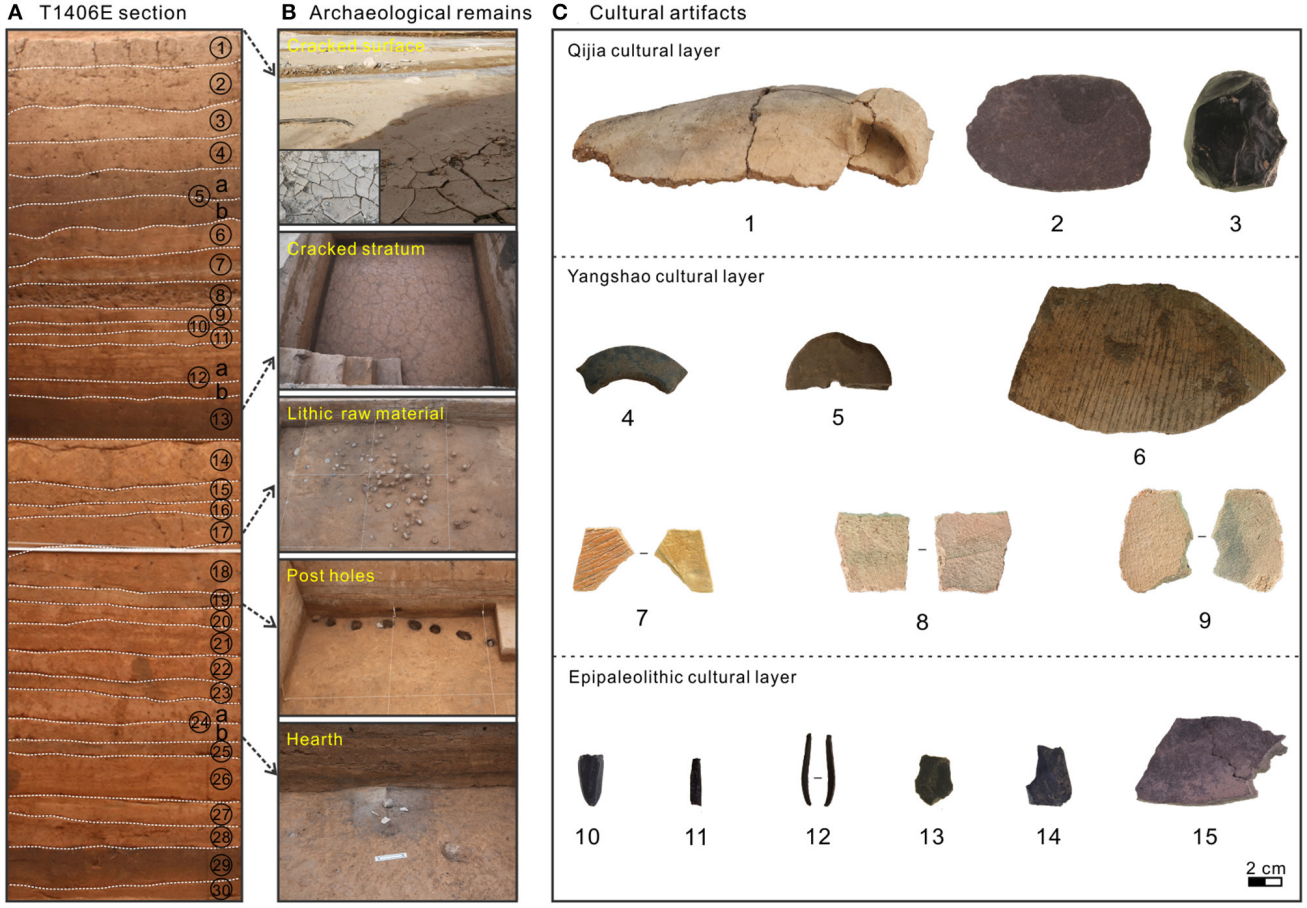
Section T1406E at Shalongka (SLK) site and typical archaeological remains and cultural artifacts from the site. **(A)** Photo of the T1406 sampling section with stratigraphic layers numbered. **(B)** Typical archaeological remains unearthed in different layers of the SLK site. **(C)** Typical cultural artifacts unearthed in different layers of the SLK site. 1. Pottery jar fragments; 2. Stone knife; 3. Core; 4. Stone ring; 5. Spinning wheel; 6–9. Yangshao cultural pottery (7: SLK-1, 8: SLK−2 and 9: SLK-3 were used for OSL dating); 10. Microblade core; 11–12. Microblades; 13: Flake; 14–15: Fragment and chunk.

Between June and August 2016, three 5 × 5 m quadrats (from south to north, termed T1404, T1405 and T1406) were excavated to a depth of ~420 cm at the SLK site by Qinghai Provincial Institute of Cultural Relics and Archaeology, with a total excavation area of 75 m^2^ (Figure 1C). Thirty stratigraphic layers were identified, some of which were further divided into sublayers (layers 5, 12 and 24), mainly based on archaeological remains, color and lithology. Layers 1–4 (0–78 cm) comprise the historical and modern cultivated layer, which is greatly disturbed by later human activity so was not analyzed in this study; layer 5 (78–90 cm) is the Qijia culture layer; layer 13 (238–268 cm) the Yangshao culture layer; and layers 17–19 (292–318 cm), 24 (345–368 cm) and 29 (388–403 cm) the Epipaleolithic culture layer (microlithic); with remaining strata comprising siltation or diluvium layers (Figures 2A,B). Preliminary field investigation showed the SLK site to be characterized by alternating deposition of cultural layers and siltation or diluvium (Supplementary Table S1).

During excavation, charcoal present in different stratigraphic layers was systematically collected for AMS ^14^C dating to establish the chronological framework; some of the dating results have been published (Wang Z. L. et al., 2021). However, charcoal dating results for the Yangshao culture layer are too young and are inconsistent with the archaeological cultural background; to better date this period, we selected three typical pottery shards (Miaodigou style) for OSL dating (Figure 2C: 7–9). We used section T1406E for environmental sampling due to its clearer and more complete stratigraphy. Overall, 208 environmental samples were collected at 2 cm intervals for analysis of GS, MS, a^*^, pollen and charcoal. In addition, four coarse sand samples from section T1406E (105, 133, 185, and 379 cm depth) and two representative floodplain sediment samples from the Yellow River and Yishaer River basins were collected for geochemical element analysis.

### ^14^C, OSL, and Collation of Published Ages

Charcoal samples were sent to the Quaternary Dating Laboratory of Peking University for AMS ^14^C dating. All dates were converted to calendar year using the IntCal 20 dataset in the Calib Rev 8.1.0 program (Reimer et al., 2020).

For OSL dating, 2–3 mm was removed from the surface of the three pottery shards (SLK1–3) under subdued red light in the lab, and the remaining material was ground in a mortar and the 90–250 μm component screened out. All samples were treated with 10% HCl and 30% H_2_O_2_ to remove carbonate and organic matter, respectively, and then etched with 40% HF for 40 min to eliminate feldspar and any potential impurities. The quartz fraction was rinsed with 10% HCl for 8 min to remove potential fluoride. Samples were rinsed repeatedly with pure water to neutralize after each step. Finally, quartz purity was detected by infrared excitation for 40 s at 125°C followed by blue excitation for 40 s at 125°C until OSL/IRSL >95%.

Equivalent doses (D_*e*_) were determined by the singlealiquot regenerative-dose (SAR) protocol (Murray and Wintle, 2000). The test instrument uses a standard Risø TL/OSL DA-20 reader with a ^90^Sr/^90^Y beta source for irradiation. Eighteen aliquots of SLK-2 and SLK-3 were measured, but only seven aliquots of SLK-1 due to smaller sample size. The environmental dose rate was determined using the beta dose in the pottery shard, along with the gamma dose and cosmic ray dose rate of surrounding soil sediments (Sun et al., 2021a). The U, Th and K content of pottery shards and sediments was obtained by inductively coupled plasma mass spectrometry (ICP-MS) and then converted into beta and gamma doses using conversion parameters (Guérin et al., 2012). The cosmic ray dose rate was calculated using the depth, elevation and geographic coordinates of each sample (Prescott and Hutton, 1994). A water content of 5 ± 2.5% was adopted, based on previous work at the SLK site (Li et al., 2014). Sample pretreatment and D_*e*_ measurements of pottery shards and sediments were carried out at the Qinghai Provincial Key Laboratory of Physical Geography and Environmental Processes, Qinghai Normal University. The environmental dose rate was measured at the Xi'an Geological Survey Center.

To augment our own dating samples, we collated previously published ^14^C and OSL ages from the formal archaeological excavation and test excavation of the SLK site (Dong et al., 2013, 2014; Li et al., 2014; Chen et al., 2015; Ren, 2017; Wang Z. L. et al., 2021). In our analysis, we mainly used chronological results from the formal archaeological excavation, due to their tight stratigraphic constraints and clear and comprehensive cultural background information; dates from the test excavation were used as for reference/support.

### Geochemistry, GS, MS, and a^*^ Analysis

Prior to pressing into discs for analysis, samples were first passed through a 200-mesh sieve to reduce the GS effect on relative element content. Elements were determined on an Axios X-ray fluorescence (XRF) spectrometer, with major element analysis uncertainties of <10%.

Samples for GS analysis were collected at 2 cm (cultural layers) and 4 cm (natural sedimentary layers) intervals. Samples were weighed to obtain test samples of 0.35 g to which 30% H_2_O_2_ and 10% HCl was added to remove organic matter and carbonate, respectively, and 10% (NaPO_3_)_6_ was then added to fully disperse the sediment (ChongYi et al., 2019). Finally, GS was determined using a Malvern Mastersizer 2000 laser particle analyzer, with an analytical range of 0.02–2,000 μm. We used end-member modeling analysis (EMMA) and mean GS parameters to gain better understanding of the sedimentary environment of the site. EMMA uses AnalySize loaded in Matlab software to perform non-parametric analysis of the original GS data (Paterson and Heslop, 2015) to describe the entire GS dataset as a mixture of unique unimodal or polymodal subpopulations (Weltje, 1997).

Samples for MS and a^*^ were collected at 2 cm intervals from the section. MS was measured on 10 g of ground, dried sediment with a Bartington MS2B sensor (470 Hz) (ChongYi et al., 2019). The a^*^ was determined on a Konica Minolta CM-2500c spectrophotometer, using the CIE standard and a^*^ denotes the red–green chromaticity. The GS, MS and a^*^ measurements were determined at the Qinghai Provincial Key Laboratory of Physical Geography and Environmental Processes, Qinghai Normal University, and geochemical analysis was performed at the Qinghai Institute of Salt Lakes, Chinese Academy of Sciences.

### Pollen and Charcoal Analysis

A total of 66 samples were selected from section T1406E for pollen and charcoal analysis, at 2 cm intervals (cultural layer) and 4–8 cm intervals (natural sedimentary layer). Standard preparation methods were used to chemically treat 50 g (dry weight) of each sample (Fægri and Iversen, 1989). Samples were boiled in 10% HCl and 10% KOH to dissolve calcareous minerals and humic components, respectively. They were then sieved through a 200-μm mesh screen and treated with 40% HF to digest fine silica, and then sieved through a 7-μm mesh to remove clay sized particles. Finally, samples were stored and mounted in glycerin jelly. Pollen and charcoal were identified at × 400 and × 1,000 magnification.

Pollen morphotypes were based on comparisons with descriptions and illustrations in Wang et al. (1997). Charcoal was counted and divided into 20–50 μm and >50 μm size groups (Tan et al., 2018; Wei et al., 2020). Approximately 300 terrestrial pollen grains and more than 800 charcoal particles were counted per sample and expressed as percentages of the total sum. Pollen and charcoal concentration (CC) was calculated based on *Lycopodium* spore tablets (27,637 ± 563 spores) that were added to the test samples, and pollen diagrams were created by using Tilia/Tilia-Graph software (Grimm, 2011). Preprocessing and identification was undertaken at the Qinghai Provincial Key Laboratory of Physical Geography and Environmental Processes, Qinghai Normal University.

## Results

### Chronology

**Figure 3:**
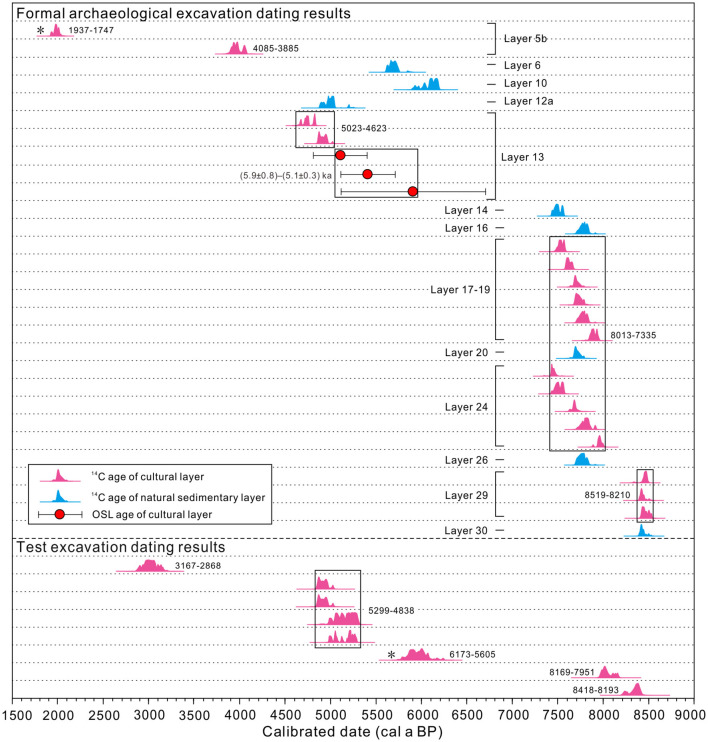
Calibrated radiocarbon and OSL age distributions from Shalongka site (Details of the age are given in Table 1 and Supplementary Table S2). *Indicates that the dating results are inconsistent with the archaeological cultural record.

Twenty-six AMS ^14^C ages (18 from the cultural layer, of which 9 are from this study) and three OSL ages for the SLK site are listed in Table 1, Supplementary Table S2 and shown in Figure 3. The dating results indicate that the age range of the SLK site is 8.5–3.9 cal ka BP. Specifically, the age of the Epipaleolithic culture layer is 8.5–7.3 cal ka BP, which can be divided into early (8.5–8.2 cal ka BP, layer 29) and late (8.0–7.3 cal ka BP, layers 24, 19, 18, and 17) stages. OSL dating of pottery shards from the Yangshao cultural layer (layer 13) gives ages of 5.9 ± 0.8–5.1 ± 0.3 ka, while the ^14^C age of charcoal in this layer is 5.0–4.6 cal ka BP; the latter is inconsistent with the OSL dating results and the archaeological cultural background, the specific reasons for which are discussed later. The ^14^C age of the Qijia culture layer (layer 5) is 4.1–3.9 cal ka BP, after exclusion of one age (BA 161048) that is much younger than the archaeological cultural background.

**Table 1:** OSL dating results of samples from the Yangshao culture layer at Shalongka (SLK) site.

**Sample ID**	**Depth (cm)**	**Sample type**	**U (ppm)**	**Th (ppm)**	**K (%)**	**Beta (Gy/ka)**	**Gamma (Gy/ka)**	**Cosmic ray (Gy/ka)**	**Water content (%)**	**Dose rate (Gy/ka)**	**Disc**	**De (Gy)**	**Age (ka)**
SLK-1	210	Pottery shard	3.71 ± 0.40	14.25 ± 0.80	2.74 ± 0.04	2.96 ± 0.04	1.56 ± 0.004	0.92	5 ± 2.5	4.12 ± 0.18	7	24.21 ± 3.06	5.9 ± 0.8
SLK-2	210	Pottery shard	4.23 ± 0.50	19.06 ± 0.90	3.24 ± 0.04	3.43 ± 0.04	1.77 ± 0.005	0.92	5 ± 2.5	4.45 ± 0.20	18	23.85 ± 0.56	5.4 ± 0.3
SLK-3	210	Pottery shard	2.50 ± 0.30	12.81 ± 0.70	2.37 ± 0.04	2.59 ± 0.04	1.44 ± 0.003	0.92	5 ± 2.5	3.7 ± 0.16	18	18.92 ± 0.47	5.1 ± 0.3
SLK-sed-1	210	Sediment	3.65 ± 0.40	13.49 ± 0.80	2.07 ± 0.04	2.42 ± 0.04	1.38 ± 0.004	0.92	–	–	–	–	–
SLK-sed-2	210	Sediment	3.38 ± 0.40	9.49 ± 0.60	2.01 ± 0.04	2.32 ± 0.04	1.29 ± 0.004	0.92	–	–	–	–	–

*The gamma dose of SLK-1 comes from SLK-sed-1; gamma doses of SLK-2 and SLK-3 were derived from SLK-sed-2*.

### Element Content

**Figure 4:**
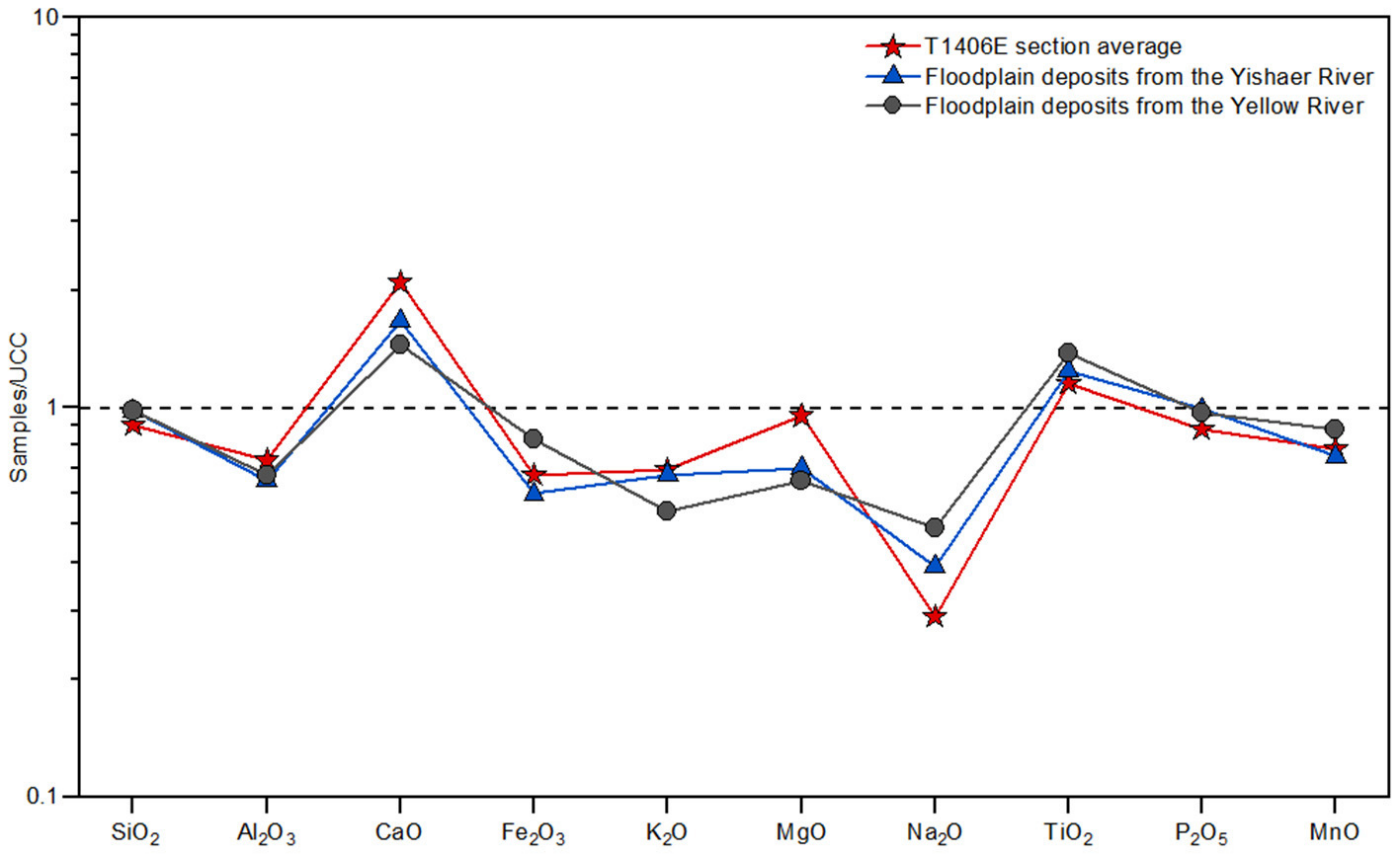
Upper continental crust (UCC)-normalized abundances of the major elements in coarse sand sediments from section T1406E and nearby modern floodplain deposits of the Yellow and Yishaer Rivers.

The elemental content of sediment samples from T1406E, Yellow River and Yishaer River basin are listed in Table 2 and shown in Figure 4. All samples are dominated by the oxides SiO_2_, Al_2_O_3_, Cao, Fe_2_O_3_, K_2_O, MgO, and Na_2_O, which comprise >86%, while the content of TiO_2_, P_2_O_5_, and MnO is <0.7%. Compared with Upper Continental Crust (UCC) (Taylor and McLennan, 1985), the content of SiO_2_ and P_2_O_5_ is close to UCC; CaO and TiO_2_ are enriched, while Al_2_O_3_, Fe_2_O_3_, K_2_O, MgO, Na_2_O and MnO are depleted.

**Table 2:** Elemental compositions of sediment samples from section T1406E at Shalongka site (SLK), and Yishaer River (YSR) and Yellow (Y) River floodplains.

**Sample ID**	**Depth**	**SiO_**2**_**	**Al_**2**_O_**3**_**	**CaO**	**Fe_**2**_O_**3**_**	**K_**2**_O**	**MgO**	**Na_**2**_O**	**TiO_**2**_**	**P_**2**_O_**5**_**	**MnO**	**Ba**	**Sr**	**Zr**	**Rb**	**Cr**	**Zn**	**Ni**	**Nb**
	**(cm)**	**(%)**	**(%)**	**(%)**	**(%)**	**(%)**	**(%)**	**(%)**	**(%)**	**(%)**	**(%)**	**(ppm)**	**(ppm)**	**(ppm)**	**(ppm)**	**(ppm)**	**(ppm)**	**(ppm)**	**(ppm)**
SLK-105	105	56.73	10.84	10.00	3.05	2.16	2.08	1.21	0.61	0.15	0.06	567.60	253.39	186.49	121.73	79.10	47.21	20.24	10.59
SLK-133	133	56.69	11.59	9.44	3.29	2.29	2.24	1.12	0.61	0.15	0.07	578.13	249.84	196.61	115.19	85.04	47.69	23.35	11.79
SLK-185	185	62.53	10.69	7.58	2.77	2.41	1.91	1.14	0.53	0.14	0.06	617.70	217.96	126.31	129.85	85.60	40.69	19.17	9.20
SLK-379	379	60.61	11.07	8.05	2.97	2.46	2.11	1.13	0.55	0.12	0.06	566.93	226.63	169.24	127.93	78.02	42.06	20.14	10.42
YSE-1	Surface	64.80	9.91	7.03	2.70	2.28	1.55	1.51	0.62	0.16	0.06	535.24	221.20	247.43	125.41	99.04	45.28	15.77	9.91
Y-1	Surface	64.91	10.23	6.08	3.72	1.82	1.43	1.93	0.69	0.15	0.07	392.63	206.15	406.85	85.92	99.61	48.29	17.35	10.17

### GS

**Figure 5:**
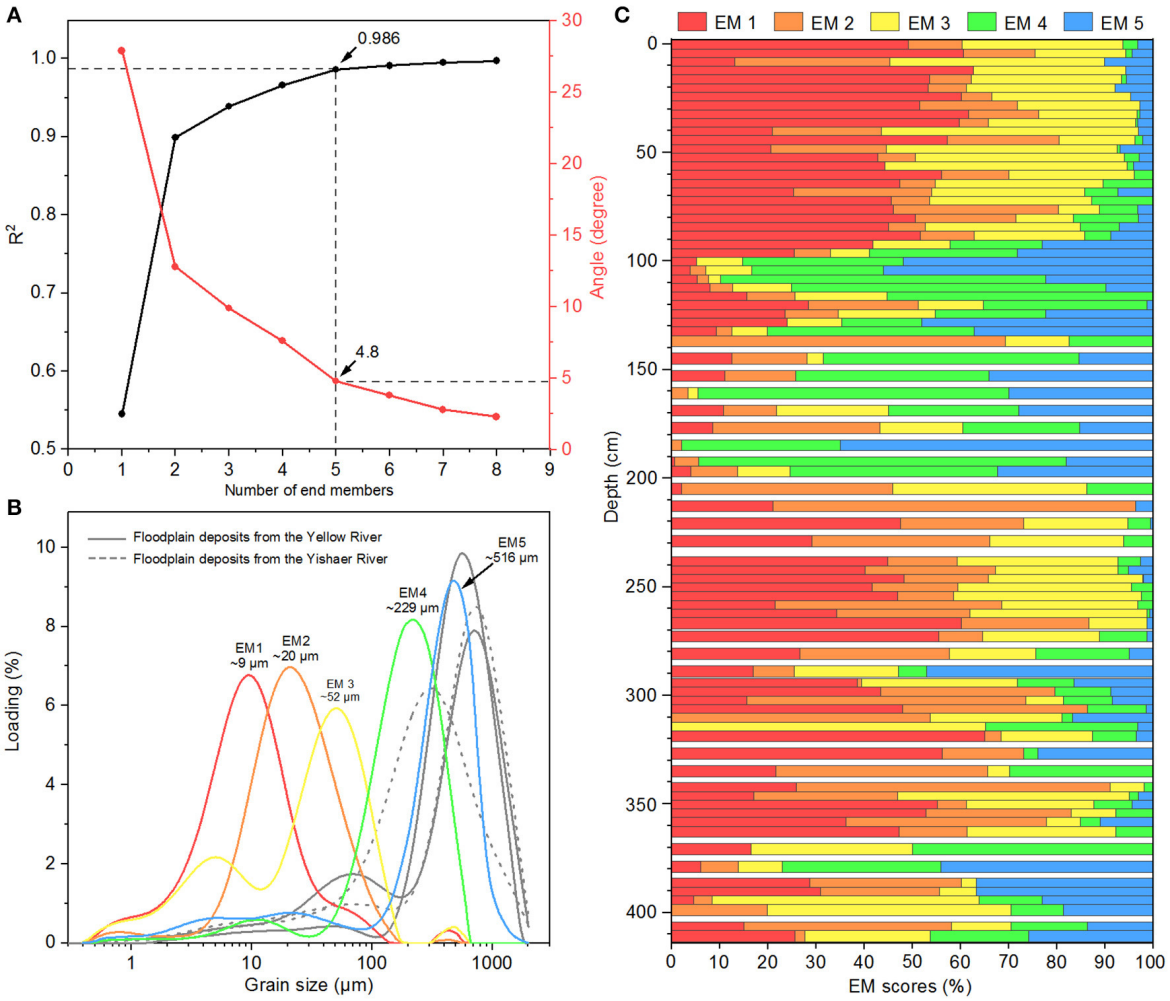
Results of end-member modeling analysis (EMMA) for section T1406E and grain size (GS) frequency distribution curves for typical floodplain sediments. **(A)** Coefficients of determination (*R*^2^) and angular deviation of EMMA; **(B)** GS frequency distribution curve of End-Member (EM)1–5, and typical modern floodplain sediments of the Yellow River and Yishaer River; **(C)** Proportions of EM1–5.

The EMMA results for section T1406E show a clear inflection at five end-members (EMs), and an angular deviation of 4.8°. The coefficient of determination (R^2^) is 0.986, indicating that the model explains 98.6% (>95%) of the total variance of the original GS dataset; R^2^ increases only slightly when EM >5 (Figure 5A). Based on the principles of parsimony and reproducibility (Weltje, 1997; Paterson and Heslop, 2015), five EM (EM1–5) were identified (Figure 5B). EM1 represents the finest component, in the fine silt range with a main mode at 9 μm; EM2 is in the medium silty range, with the main modal at 20 μm; EM3 is in the coarse silt range and presents a bimodal distribution, with the main peak at ~52 μm (coarse silt) and a secondary peaks ~5 μm (fine silt); EM4 is in the fine sand range and is poorly sorted, with a main peak of ~229 μm; EM5 represents the coarsest component, in the coarse sand range, and is poorly sorted, with the main peak at ~516 μm.

EM scores are shown in Figure 5C. For EM1, the score ranges from 0 to 65.1%, with an average of ~31.0%. EM2 score ranges from 0 to 75.2%, with an average of ~19.2%. EM3 score ranges from 0 to 65.3%, with an average of ~22.1%. EM4 and EM5 show similar trends, with scores of 0–76.4% and 0–64.9%, respectively, averaging 15.4% and 12.4%. Below 270 cm, the scores for EM4 and EM5 are relatively high and fluctuate greatly, with averages of 12.7 and 13.5%, respectively; the highest scores are 210–90 cm, with averages for EM4 and EM5 of 38.5 and 25.6%, respectively.

**Figure 6:**
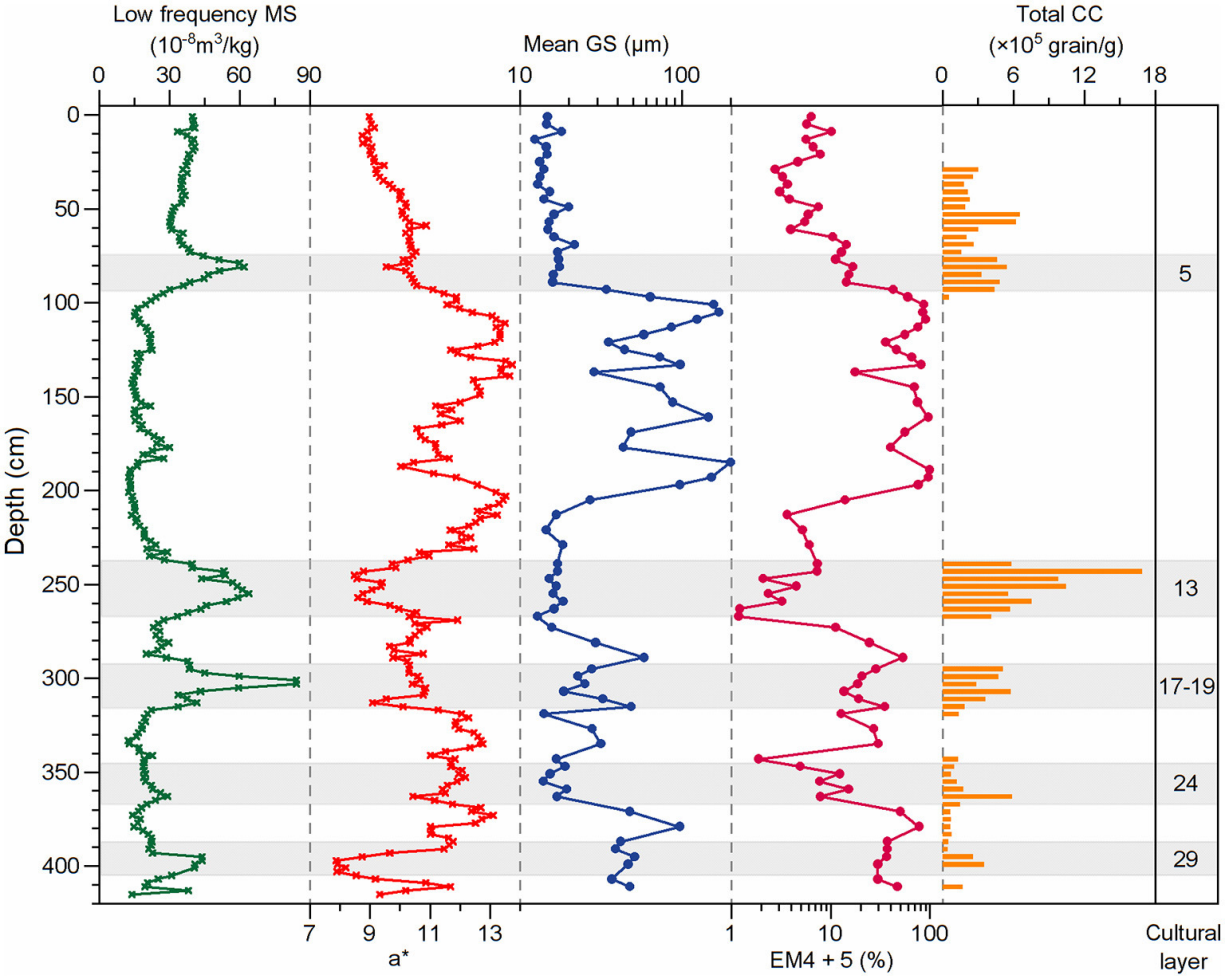
Variations in environmental proxies in section T1406E. Symbols and terms on the graphs are defined as: MS, magnetic susceptibility; a*, redness; GS, grain size; EM, end-member; CC, charcoal concentration.

Mean GS show significant variation in section T1406E, ranging from 12.3 to 198.4 μm, with an average of 39.7 μm (Figure 6). Based on GS results, the section can be divided into four zones: (1) 415–292 cm, with mean GS range of 13.8–96.2 μm and average of 33.7 μm; (2) 292–210 cm, with a relatively stable mean GS range of 12.7–29.0 μm and average of 17.1 μm; (3) 210–90 cm, with mean GS range of 28.5–198.4 μm and average of 90.1 μm; (4) 90–0 cm, with the lowest mean GS range of 12.3–21.5 μm and average of 15.5 μm.

### MS and a^*^

Trends in a^*^ and low frequency MS in section T1406E are roughly inverse (one increases as the other decreases), but a^*^ is more sensitive (Figure 6). a^*^ ranges 7.9–13.7, with an average of 11.0; low frequency MS between 12.3–84.3 × 10^−8^ m^3^/kg, with an average of 28.3 × 10^−8^ m^3^/kg. a^*^ is generally >11.0 in the layers at 411–407, 393–318, 292–268, and 231–97 cm, while low frequency MS is <27 × 10^−8^ m^3^/kg.

### Pollen and Charcoal

**Figure 7:**
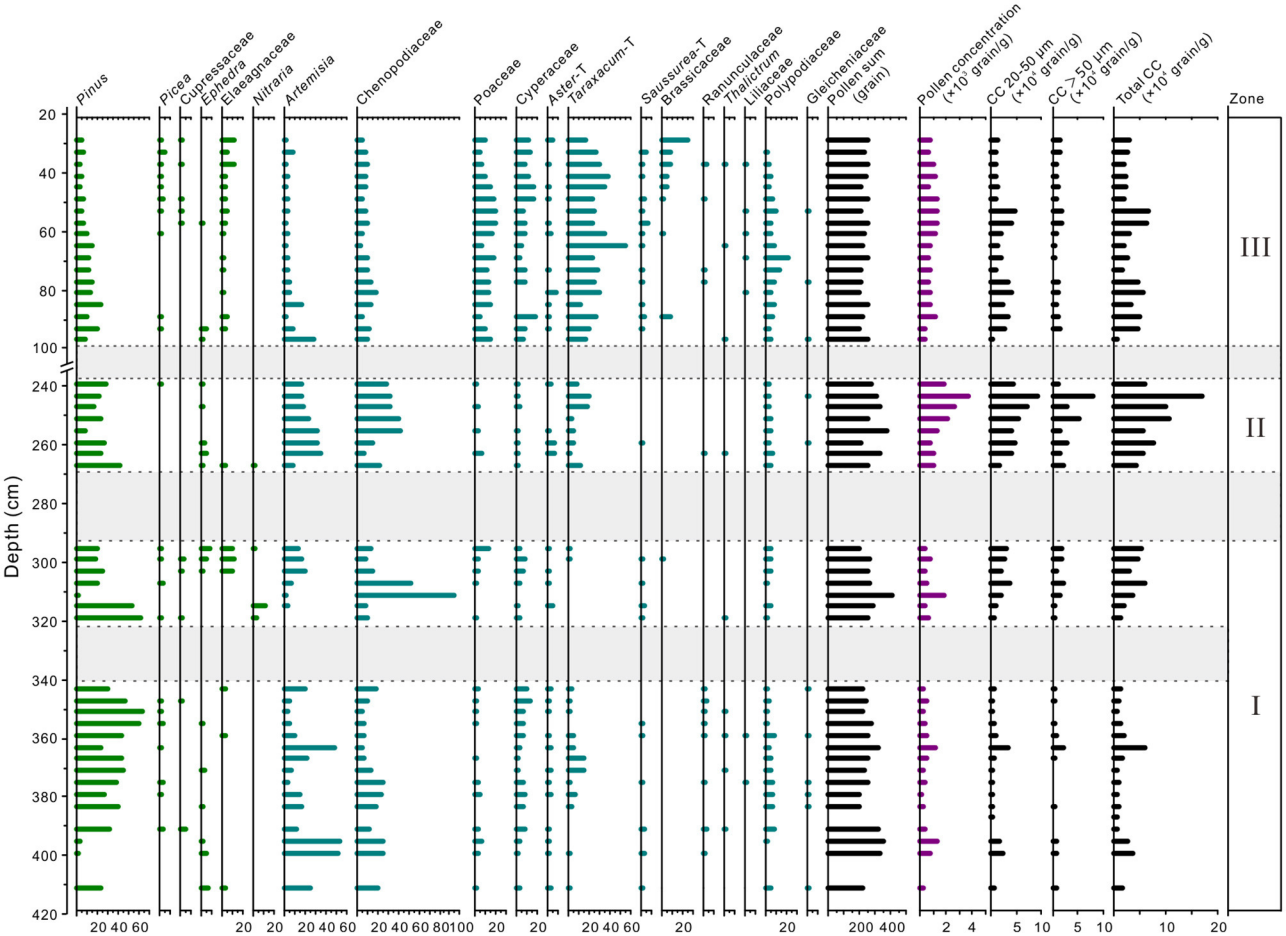
Percentage of main pollen taxa and charcoal concentration (CC) at section T1406E.

No or little pollen and charcoal was found in the intervals 342–318, 292–268, and 238–97 cm of section T1406E due to the influence of sedimentary environment. Overall, 49 pollen taxa were identified in the 48 samples from the section. Pollen taxa mainly include *Pinus, Picea*, Cupressaceae, *Carpinus, Larix, Quercus, Ephedra*, Elaeagnaceae, *Nitraria, Artemisia*, Chenopodiaceae, Poaceae, Cyperaceae, Asteraceae, Brassicaceae and Ranunculaceae. Based on variations in pollen assemblage, CC and sediment lithology, three zones were identified (Figure 7). Zone I (411–342 and 318–292 cm) corresponds to layers 29, 24, and 19–17, indicating the Epipaleolithic age cultural layer. Zone II (268–238 cm) corresponds to layer 13, indicating the Yangshao cultural layer. Zone III (90–20 cm) corresponds to layers 5–3, of which five (90–78 cm) are Qijia culture.

Zone I: Pollen assemblages are dominated by *Pinus* (range 2.1–64.9%, average 33.3%, the same below), Chenopodiaceae (4.5–93.4, 19.3%), *Artemisia* (0.8–54.3, 16.5%) and Cyperaceae (0.5–13.0, 5.5%), with a small amount of Polypodiaceae (0.3–8.0, 4.0%), *Taraxacum*–type (0–15.0, 3.0%), Poaceae (0.2–13.4, 2.9%), Elaeagnaceae (0–11.8, 2.2%), and *Picea* (0–4.0, 1.7%). The pollen concentration is 515.5 grains/g. The total CC is 22,258 grains/g, with that >50 μm and 20–50 μm being 8,161 and 14,097 grains/g, respectively. CC of >50 μm and 20–50 μm is generally low in the substage at 411–342 cm, except for peaks at 399 cm and 363 cm, while in the substage at 320–290 cm CC gradually increases to 35,908 grains/g.

Zone II: The percentage of Chenopodiaceae (7.3–42.4, 27.8%), *Artemisia* (8.6–34.6, 22.9%), and *Taraxacum*–type (2.1–19.6, 9.0%) increases significantly, while that of *Pinus* (8.5–43.0, 24.1%), Cyperaceae (0.4–2.7, 1.5%), and *Picea* (0–2.9, 0.9%) decreases significantly. Overall pollen concentration in this zone increases significantly (1,761 grains/g). Total CC reaches a maximum in this zone, with 81,997 grains/g. Compared with zone I, CC of >50 μm and 20–50 μm is significantly higher, with 30,716 and 51,282 grains/g, respectively, and total CC reaches a maximum of 168,557 grains/g at 243 cm.

Zone III: The pollen assemblage is characterized by a significant decrease in the percentage of *Pinus* (3.4–23.1, 10.5%), Chenopodiaceae (3.6–18.3, 9.6%), and *Artemisia* (1.4–27.8, 5.7%), and increase in *Taraxacum*–type (12.5–55.7, 27.5%), Poaceae (4.6–21.1, 13.1%), Cyperaceae (0.4–19.0, 9.1%) and *Picea* (0–5.2, 1.6%). Overall pollen concentration is relatively low (852.8 grains/g). Total CC decreases significantly (32,409 grains/g), and that of >50 μm and 20–50 μm decreases to 10,001 and 22,408 grains/g, respectively. However, there are peaks in the CC of 20–50 μm ~at 93–77 and 53 cm.

## Discussion

### Reconstruction of Depositional Environment at the SLK Site

As mentioned in Section Field Work and Sampling, field investigations suggest that development of SLK site has been affected by aeolian and fluvial deposition, but the provenance of floodplain sediments at the site is still unclear. SLK site is located close to the junction of the Yellow River and its tributary the Yishaer River, with the two rivers ~500 and 260 m from the site, respectively. Hence, both basins are potential source areas of floodplain sediments at the site.

Comparison of the geochemical characteristics of typical floodplain sediments from T1406E with those of the Yellow River and Yishaer River (Figure 4) shows the major and trace element content of the section is generally closer to those of Yishaer River. The same pattern is shown in the UCC-normalized abundances of average major element content, where sediments from T1406E and Yishaer River have similar trends of relative enrichment/depletion that differ from Yellow River sediments (excluding SiO_2_, P_2_O_5_, and Al_2_O_3_ where the three samples overlap). In addition, the common presence of poorly rounded gravels in the coarse sand layer of section T1406E indicates a short transportation distance. Thus, several strands of evidence support the provenance of floodplain sediments at SLK site being the Yishaer River basin rather than the Yellow River basin.

Separation of aeolian and fluvial sediments at the site is important for understanding the paleoenvironmental background of prehistoric human activities. We used EMMA to decompose the GS dataset from section T1406E into two components sensitive to aeolian and fluvial processes.

Vandenberghe (2013) reviewed the relationship between GS distribution and facies of fine-grained windblown sediment, identifying several loess populations that may help disentangle the provenance of our sediments. The modal size of EM1 (~9 μm) in our study is similar to Vandenberghe's (2013) subgroup 1.c.2 (mode 4 μm, range 2–10 μm) that is common in loess of the northeastern QTP and Tajikistan. This component is considered to be transported by long-term high-level westerlies (Nottebaum et al., 2015). The modal size of EM2 (~20 μm) is similar to Vandenberghe's (2013) subgroup 1.c.1 (mode 19 μm, range 16–22 μm) that common in loess of the CLP (Prins et al., 2007; Vriend, 2007). This is a relatively stable component of background atmospheric dust.

The modal size of EM3 (~52 μm) corresponds to Vandenberghe's (2013) 1.b.1 component (51–60 μm) that appears in loess of the northeastern QTP and the CLP (Vriend and Prins, 2005) and is considered to be a dust storm component from proximal sources (Vriend et al., 2011). However, considering the SLK site is in the lower elevation area of the southeastern Qunjian Basin and close to a major river junction, it can widely accumulation aeolian dust from far and near sources. Thus, we infer that EM1–3 is probably of aeolian sedimentary origin, but its relatively high clay and sand content indicates that it may have been subject to post-depositional reworking. Therefore, field investigation and other proxy evidence is needed to further clarify the specific processes.

The major peaks of EM4 and EM5 are at 229 and 516 μm, respectively, with a small mixed component of clay and fine silt. The GS distribution frequency curves for these EMs are similar to those of modern floodplain sediments in the Yellow River and Yishaer River basins (Figure 5B). Moreover, previous studies show that fluvial sediments are usually composed of medium sand saltation components (200–400 μm) and fine silt suspension components (10–15 μm) (Pye and Tsoar, 1987; Bennett and Best, 1995). Therefore, EM4 probably reflects a lower energy floodplain environment and EM5 a higher energy floodplain environment. These results are also similar to the GS distribution patterns of coarse and fine components of sediments in Heihe and Shiyang River Basins and Hanzhong Basin, China (Yang et al., 2019; Wang B. L. et al., 2021). Based on this, we use the sum of EM4 and EM5 as an index to indicate the strength of hydrodynamic conditions at the SLK site.

To further clarify the impact of aeolian and hydrologic processes at SLK site, low frequency MS and a^*^ were used as supplementary indicators of the sedimentary environment of the site. MS refers to the relative concentration of ferromagnetic minerals in sediments and is an index of the intensity of weathering and pedogenic modification of aeolian sediments of the northeastern QTP and the CLP; MS is usually between (28–100) × 10^−8^ m^3^/kg (Huang et al., 2013; Xue and Zeng, 2021), reflecting the intensity of the EASM (Kang et al., 2020). However, some magnetic minerals are subject to dissolution under the reducing conditions of shallow incubation (shallow marine or lake sediments), resulting in an overall decrease in low frequency MS (Ao et al., 2010; Peng et al., 2021). Considering the complexity of the sedimentary environment at SLK site, it is reasonable to infer that low frequency MS is relatively high when aeolian processes are dominant, and relatively low when hydrogenesis is dominant.

a^*^ is mainly controlled by the type and concentration of iron oxide minerals, especially hematite (Ji et al., 2005; Sun et al., 2020). Previous work suggests that a^*^ is related to river action on the QTP: Ji et al. (2005) linked a^*^ with river-transported terrestrial materials in the lacustrine sediments of Qinghai Lake on the northeastern QTP, reflecting regional monsoon and precipitation changes. Brownish red argillaceous rock and brick red fine-grained rock are widely distributed in the northern area of the SLK site. Moreover, the alluvial and proluvial effect of floodplain facies is obvious after preliminary field investigation and consulting the 1:250,000 geological map (http://www.ngac.cn/125cms/c/qggnew/index.htm). Combined with our geochemical and EMMA results, we conclude that high a^*^ reflects material that is weathered and eroded from nearby red beds or loess deposits and is transported to the site by river flow under high energy conditions of Yishaer River.

Figure 6 shows high EM4+5 scores correspond with high a^*^ and low MS, which is supports a fluvial sedimentary origin for EM4+5. EM1–3 is mainly related to the post-depositional reworking of river and lake deposits under the influence of aeolian sedimentation. Therefore, EMMA results, low frequency MS and a^*^ of section T1406E provide good supplementary sedimentary environment indicators for the SLK site.

Based on stratigraphic trends in the environmental indicator results in section T1406E (Figure 6), four stages in the evolution of the sedimentary environment at the SLK site can be identified. In the lower part of the section (411–268 cm), the EM4+5 score, mean GS and a^*^ values are relatively high and fluctuate significantly, while low frequency MS is relatively low, indicating the stage represents a generally hydraulic but occasionally dry environment (403–388, 482 368–345, and 318–292 cm).

The next stage (268–238 cm) corresponds to the Yangshao cultural layer. The EM4+5 score, mean GS and a^*^ values are low and stable, while low frequency MS is high (Figure 6), indicating a switch from hydraulic to predominately aeolian sedimentation.

In the next stage (238–90 cm), a^*^ and low frequency MS show an inverse relationship, changing simultaneously with a^*^ generally high and MS generally low, while the EM4+5 score and mean GS show a sharp increase after initially low levels (Figure 6). In addition, poorly rounded small gravel is present at 182–178 cm depth. All evidence points to a strong hydrodynamic environment in this stage, driving the large increase in GS. The reduction in low frequency MS is due to the long-term underwater environment.

The final stage (90–0 cm) is characterized by relatively high low frequency MS, while the EM4+5 score, mean GS and a^*^ values are very low (Figure 6). These trends indicate reduced input of fluvially transported materials and dominance of aeolian sedimentation.

### Vegetation and Climate Background at the SLK Site

Based on the pollen record of section T1406E, we have reconstructed the vegetation and climate characteristics of SLK site in the Epipaleolithic, Yangshao culture and Qijia culture period. The arboreal pollen of T1406E is mainly *Pinus*, accompanied by a small amount of *Picea* and Cupressaceae (Figure 7). Relationships between modern pollen and vegetation suggest that *Pinus* is over-represented as it can be transported by wind and river over long distances (Miehe et al., 2014; Wei et al., 2020). *Pinus* pollen content is always below 5% in the desert areas of eastern China, and often exceeds 30% in *Pinus* forest (Li et al., 2005). In section T1406E, *Pinus* percentages fluctuate between 10.5 and 33.3%, which suggests pine forest was developed on mountains near to the SLK site.

The non-arboreal pollen taxa of section T1406E consist mainly of *Artemisia*, Chenopodiaceae, Cyperaceae, Poaceae and Asteraceae (Figure 7). Previous studies of surface pollen on the QTP suggest that *Artemisia*, Asteraceae and Poaceae are the most important taxa of the temperate steppe (Lu et al., 2011; Wei et al., 2011, 2020), Chenopodiaceae is the dominant taxa of temperate desert steppe/desert vegetation (El-Moslinmny, 1990; Zhao and Herzschuh, 2009), while Cyperaceae dominates in alpine meadow and meadow-steppe (Lu et al., 2011; Wei et al., 2018).

The EMMA analysis results show that Zone I (411–342 and 318–292 cm) is affected by the fluvial action of Yishaer River, so the pollen sources for section T1406E comprise that transported by river and wind and local sites. Since the SLK site is only ~4 km from the piedmont of Laji mountain, and the pollen transport distance is relatively local, the pollen records likely reflect vegetation conditions in areas adjacent to the site. The high content of *Pinus*, Chenopodiaceae and *Artemisia* in Zone I, indicates that forest-steppe vegetation is developed around the site (Figure 7). The low content of Cyperaceae and Polypodiaceae indicates relatively humid hydrological conditions, pointing to a relatively warm and humid regional climate in this stage. Zooarchaeological studies have identified the remains of small deer in cultural layer 19–17 (Yi et al., 2020), which usually live at the forest-steppe boundary (Zhang et al., 2015), further confirming the vegetation reconstruction results.

Zone II (268–238 cm) is dominated by aeolian deposits, and the pollen assemblage is characterized by Chenopodiaceae, *Pinus, Artemisia* and Asteraceae (Figure 7), in which the *Pinus* content (mean of 24.1%) is still relatively high. This generally reflects forest-steppe vegetation with relatively reduced arboreal coverage, and a relatively warm and humid climate.

Zone III (90–20 cm) is also dominated by aeolian deposits. Vegetation is dominated by Asteraceae, Poaceae and Cyperaceae, accompanied by stable and low content of Elaeagaceae, while the content of Chenopodiaceae, *Artemisia* and *Pinus* decreases significantly (Figure 7), indicating development of a sparse forest-shrub-steppe landscape and a relatively cool and dry climate.

### The Relationship Between Environment Change and Human Occupation at the SLK Site

Social productivity was low in the prehistoric period. For survival, people first needed to choose a suitable production and living environment, and this largely depended on finding a suitable geomorphic environment. During the early Epipaleolithic (403–388, 368–345, and 318–292 cm, layers 17–19, 24, and 29, 8.5–8.2 cal ka BP and 8.0–7.3 cal ka BP) human occupation of the SLK site, the EM4+5 scores (especially EM5), mean GS and a^*^ values are relatively low (Figure 6), indicating it was a period with less hydrogenesis.

During the Yangshao culture period of the Neolithic (268–238 cm, layer 13), the SLK site was subject to stable geomorphic conditions and land resources. Archaeologists ascribed this layer as the Miaodigou style of Yangshao culture, dating to 6.0–5.1 cal ka BP, based on typology of archaeological remains and artifacts unearthed (Qinghai Provincial Institute of Cultural Relics and Archaeology, 2013; Chinese Society of Archaeology, 2017; Han, 2021). However, ^14^C dating of charcoal from layer 13 gives a younger age of 5.0–4.6 cal ka BP (Wang Z. L. et al., 2021), which is inconsistent with the archaeological cultural background. The difference in ages may be related to the site micro geomorphology and/or disturbance. As shown in Figure 6, upper part (245–238 cm) of the Yangshao cultural layer, the EM4+5 score increased moderately, a^*^ increased significantly and low frequency MS decreased rapidly. Also, survey of the layer by Electronic Total Station shows that the perimeter area is ~30 cm higher than the center, indicating a shallow basin/hollow. Archaeological excavation confirmed the presence of cracks in the stratum surface, similar to desiccation cracks on the modern floodplain of the Yishaer River that appear after pools of standing water have dried out (Figure 2B).

The above evidence suggests that the Yangshao cultural layer is in a low-lying area that was generally affected by aeolian sediments, while the upper part (245–238 cm) of the layer was also subjected to slight fluvial transportation and slope clastics from local areas, which may affected the distribution of smaller and lighter articlasts during deposition. Therefore, there is a good probability that the charcoal material used to date the Yangshao cultural layer is associated with secondary deposition, hence resulting a younger dating results.

Recently, Sun et al. (2021a,b) applied OSL dating to prehistoric and historical pottery from sites on the northeastern QTP using OSL. The results are consistent with OSL and ^14^C dating of stratum sediments and historical records and demonstrate the reliability of OSL dating of pottery in the region. In this study, OSL dating of three typical pottery shards (Miaodigou style) from the Yangshao cultural layer gave ages of 5.9 ± 0.8–5.1 ± 0.3 ka, which is consistent with the archaeological cultural background. Hence, it confirms that prehistoric humans occupied the SLK site during this period.

No evidence of prehistoric human activities has been found between 238 and 90 cm, which the proxy evidence shows was a period of strong hydrodynamic conditions, suggesting prehistoric humans were forced to withdraw from the SLK site at this time. The overlying layer (90–78 cm) corresponds to the Qijia culture and the dating results show that prehistoric humans occupied the site between 4.1 and 3.9 cal ka BP. Both geomorphic survey and EMMA results suggest that prehistoric humans migrated to the SLK site after the flooding when river erosion had formed a stable terrace. In summary, the history of occupation and abandonment of SLK site appears to be closely related to the hydrodynamic conditions at the site. When hydrodynamic energy is high, the intensity of prehistoric human activities is weak or absent, while when hydrodynamic energy is low (i.e., aeolian conditions prevail), there is human occupation of the site.

**Figure 8:**
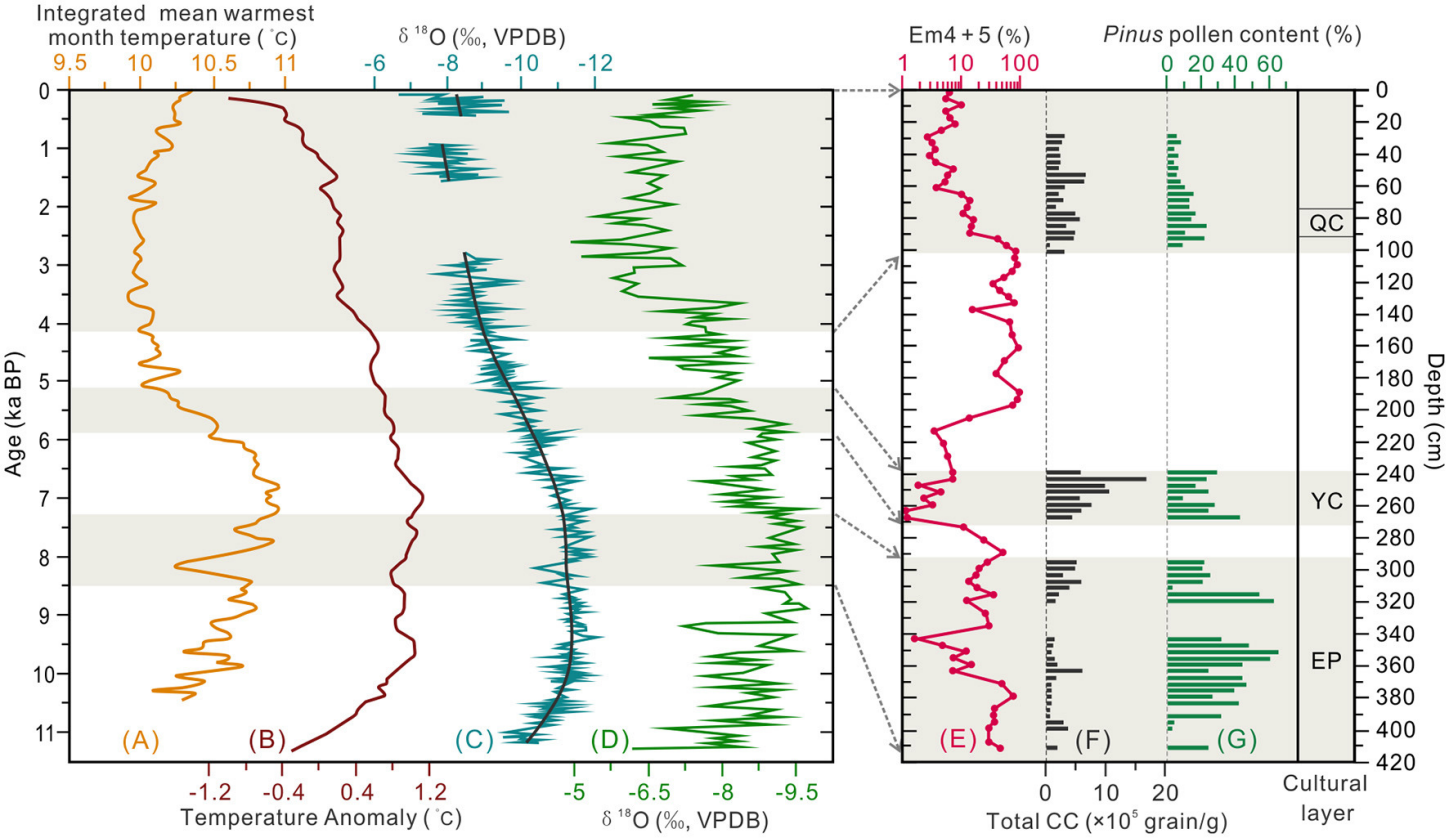
Comparison of the Environmental proxies from the section T1406E (Shalongka site) with nearby climate records. **(A)** Integrated reconstruction of Holocene mean warmest month temperature in eastern Qinghai–Tibetan Plateau (Liang et al., 2020); **(B)** Quantitative reconstruction of Holocene temperature record in the northern hemisphere (90–30°N) (Marcott et al., 2013); **(C)** Holocene monsoon changes recorded by high-resolution stalagmites on the western Chinese Loess Plateau (Tan et al., 2020); **(D)** δ^18^O record of the Asian monsoon from Dongge cave, Guizhou Province, China (Dykoski et al., 2005); **(E)** EM4 + 5 score mean grain-size of the section T1406E; **(F)** Total charcoal concentration (CC) of the section T1406E **(G)** *Pinus* pollen content of the section T1406E. EP, Epipaleolithic; YC, Yangshao culture; QC, Qijia culture.

To better understand the environmental background of prehistoric humans at the SLK site, we have collated high-resolution paleoenvironmental records for areas adjacent to the Qunjian Basin. The Holocene temperature record based on quantitative analysis of pollen records in the eastern QTP (Liang et al., 2020) indicates mean monthly temperature peaked at ~11°C around 8.5–6 ka BP (Figure 8A). An early Holocene (10.0–7.0 ka BP) temperature peak is supported by a larger scale reconstruction of temperature at middle and high elevations of the northern hemisphere by (Marcott et al., 2013, Figure 8B). Stalagmite δ^18^O records (~5-year resolution) from the western CLP (Tan et al., 2020) show the wettest period was between 10.5–6.6 ka BP (Figure 8C); combined with the pollen record for section T1406E, the data indicate that prehistoric humans of Epipaleolithic age lived in a warm and humid forest-steppe environment. During the period of the Yangshao culture, although hydrothermal conditions decreased, the EASM was still relatively strong (Figure 8D); the SLK site was in the forest-steppe with reduced arboreal coverage and relatively amenable climate environment. During the Qijia culture period, the climate background tended to be cold and dry and sparse forest-shrub-steppe was developed around the site (Figure 8).

### Regional Comparison: Survival Pattern of Human Activity at the SLK Site

Prehistoric human adaptation to environment is documented through patterns of mobility. The charcoal record is an effective proxy for climate change and human activities (Miehe et al., 2014); high CC denotes high frequency of fire events, and vice versa (Wei et al., 2020). In section T1406E, peak CC corresponds with culture layers, indicating that CC reflects the human use of fire. To document and analyze the evolution of human mobility patterns at the SLK site, we combined CC evidence with EMMA, pollen results and formal archaeological excavation materials, and compared with prehistoric human activities on the high-altitude QTP and the low-altitude Loess Plateau.

During the early Epipaleolithic (8.5–8.2 cal ka BP, layer 29), stratigraphic sedimentation is thick (~15 cm), with a small numbers of microblades and pottery fragments recovered (Figure 2C), and four small wild mammal bones (Yi et al., 2020). The relatively high EM4+5 score shows river action is ongoing (Figure 8E). Therefore, SLK site may have been a temporary campsite for short-term foraging use at this time, with people adopting a high mobility migration strategy. Total CC in section T1406E is generally low, but increases significantly at ~380 cm, indicating the first human activities at the SLK site (Figure 8F).

During the late Epipaleolithic (8.0–7.3 cal ka BP, layers 24 and 19–17), stratigraphic sedimentation is relatively thick (~49 cm), and recovered artifacts include a hearth and >400 pieces of microlithic artifacts, including microblade cores, microblades and scrapers were unearthed (Figure 2C). The assemblage is consistent with the knapped lithic techniques at sites in high elevation areas of the QTP such as Xidatan 2 (~9.2–6.4 ka BP, 4,300 m a.s.l.) (Brantingham et al., 2013), Canxionggashuo (~8.2–7.4 ka BP, 4,016 m a.s.l.) (Han et al., 2020) and Yeniugou (~7.5 ka BP, 3,800 m a.s.l.) (Tang et al., 2013). Archaeobotanical analysis identified 364 fragments, including snails, birds, small rodents, rabbits and small deer in cultural layers 19–17 at SLK site (Yi et al., 2020). Total CC increases gradually through the period, indicating the intensity of human activities continued to develop (Figure 8F).

It is worth noting that seven linearly distributed post holes have been found in the cultural layer 19 (Figure 2B). Archaeologists suggest that this may be the remains of stilt style architecture, showing that the mobility of prehistoric humans decreased significantly and tended to settlement. Suspected pottery shards were also unearthed in this layer, and the Chenopodiaceae pollen content (up to 93%) increased simultaneously with CC at ~310 cm in section T1406E (Figure 7), which implays that humans might have collected edible vegetables of Chenopodiaceae family for their food resources. This estimation is also supported by the findings of starch grain (found root tuber) in the microlithic cultural layer (Zhao et al., 2021). However, the EM4+5 score shows that the site is more weakly affected by river action in the Epipaleolithic (below 292 cm) (Figure 8E), and given the characteristics of monsoon climate in this region, prehistoric humans probably inhabited the site in winter and spring when the river was relatively dry. To sum up, it seems the SLK site was used for processing of knapped microliths and was under long-term regular use and seasonal settlement (winter and spring campsite) by people following a hunting and gathering lifestyle. Correspondingly, the mobility of prehistoric humans was significantly reduced during the late Epipaleolithic age.

During the Yangshao culture period of Neolithic age (5.9 ± 0.8–5.1 ± 0.3 ka, layer 13) stratigraphic sedimentation is ~30 cm. The formal archaeological excavations in 2003 and 2016 identified 19 houses sites, with a large number of pottery and stone rings unearthed during for period (Qinghai Provincial Institute of Cultural Relics and Archaeology, 2013; Chinese Society of Archaeology, 2017). Further studies on starch grains showed that ancient humans used *Setaria italica* (Zhao et al., 2021). This is very different to the microlithic sites found in the previous or same period on the QTP (Brantingham et al., 2007; Hou et al., 2016), indicating that a new cultural type from the Yellow River basin has spread to the SLK site. Meanwhile, genetic studies show that the main population of modern settlers on the QTP came from the Yangshao culture population in the middle and upper reaches of the Yellow River at ~6.0 ka BP (Zhao et al., 2009); linguistic studies also show that initial splitting of the primitive Sino Tibetan language occurred ~5.9 cal ka BP (Zhang et al., 2019). These ages are consistent with the OSL dating results (5.9 ± 0.8 ka) for Yangshao cultural layer pottery shards in this study. Based on the above multidisciplinary evidence, we believe that the SLK site probably represents the earliest Neolithic population spreading from the middle reaches of the Yellow River to the northeastern QTP.

Another significant feature of the Yangshao culture layer is the large number of microliths, including microblade cores, microblades and scrapers that have been unearthed, along with >200 bone artifacts, including fish hooks and needles. These finds far exceed that of Yangshao culture sites (Miaodigou style) at lower elevations in the middle and lower reaches of the Yellow River (Qinghai Provincial Institute of Cultural Relics and Archaeology, 2013; Xiao, 2013). Considering that the tradition of microlithic technology previously prevailed at SLK site, and human activities in high elevation areas of the QTP are still dominated by microlithic technology in the period and later (Gao et al., 2020; Zhang X. L. et al., 2020; Chen et al., 2021), it is inferred that the SLK site is probably also occupied by microlithic hunter-gatherers indigenous to the QTP at this stage. Total CC increased to its highest level in the section T1406E, indicating enhanced intensity of human activities and settlement of the site (Figure 8F).

To sum up, the earliest Neolithic culture on the QTP results from interactions between Neolithic millet farmers introduced from the CLP and indigenous microlithic hunter-gatherers. At this stage, prehistoric humans not only engaged in relatively stable agricultural production, but also in mobile hunting-gathering activities. There are two main reasons for this survival strategy. First, as the site is less affected by flooding (EM4+5 score is very low) (Figure 8E), there are stable geomorphic conditions for agricultural development. Second, in order to reduce the risks brought by the natural environment—river junction location—prehistoric humans took the initiative to adopt millet agriculture technology, so as to maximize the use of diversified food resources in the forest-steppe ecological ecotone (Figures 7, 8G).

During the Qijia culture period, stratigraphic sedimentation was ~12 cm, in which house sites, post holes, ash pits and ash ditches have been found (Chinese Society of Archaeology, 2017). Zooarchaeological studies have found remains of pig, cattle and goat that may be domesticated, as well as wild animals such as small deer (Yi et al., 2020). Archaeobotanical studies has found a large amount of millet, as well as wheat and barley from west Asia (Yi et al., 2020; Zhao et al., 2021). It can be concluded that prehistoric humans adopted a novel agropastoral and hunting economy, forming a diversified lifestyle dominated by settlement. The subsistence strategies could represent active adaptation of prehistoric humans to the increasingly cold and dry climatic (Figure 8). Total CC has decreased compared with the Yangshao culture layer (Figure 8F), which may be related to a decrease in biomass due to vegetation degradation and human conscious fire management.

As an important transition area between the QTP and the CLP, the SLK site exhibits ecological characteristics of both high and low elevation regions, and is transitional in terms of climate. Therefore, in the Epipaleolithic to Neolithic transition, the area may play an important role in the communication and integration of different people and cultures.

## Conclusion

Multi-proxy records from section T1406E show that the SLK site was affected by the local fluvial sedimentary environment, the hydrodynamic energy of cultural layers is small or non-existent. The absolute dating results at the SLK site reveals that prehistoric humans occupied the site during the Epipaleolithic (8.5–7.3 cal ka BP), Yangshao culture (5.9–5.1 ka) and Qijia Culture (4.1–3.9 cal ka BP) periods, respectively. Humans lived a landscape that was predominated by forest-steppe, and the forest coverage gradually decreased from early to late period. Comprehensive multidisciplinary evidence shows that the Epipaleolithic was occupied by hunter gatherers, and transitioned from high mobility in the early stage to relatively fixed seasonal central campsite in the late Epipaleolithic. During the Yangshao culture period, farmers from the CLP spread to upper Yellow River valleys on the QTP, forming the earliest settled village on the QTP (~5.9 ka). During the Qijia culture period, prehistoric humans adopted a diverse use of natural resource overcome the cold and dry climate environment.

## Data Availability Statement

The original contributions presented in the study are included in the article/Supplementary Material, further inquiries can be directed to the corresponding author/s.

## Author Contributions

GH and JG conceived and designed the study. GH, JG, YX, ZW, SJ, and XC carried out field work. JG, GH, HW, CE, YS, HX, and MS analyzed data. JG wrote the first draft of the manuscript. JG, GH, HW, and ZW revised the manuscript. All authors contributed to the article and approved the submitted version.

## Funding

This study was supported by the National Natural Science Foundation of China (Grant No. 42171165), the Strategic Priority Research Program of the Chinese Academy of Sciences, Pan-Third Pole Environment Study for a Green Silk Road (Pan-TPE) (Grant No. XDA2004010101), and the Qinghai Provincial Project for Thousand Top Innovative Talents (Grant No. 2019006).

## Conflict of Interest

The authors declare that the research was conducted in the absence of any commercial or financial relationships that could be construed as a potential conflict of interest.

## Publisher's Note

All claims expressed in this article are solely those of the authors and do not necessarily represent those of their affiliated organizations, or those of the publisher, the editors and the reviewers. Any product that may be evaluated in this article, or claim that may be made by its manufacturer, is not guaranteed or endorsed by the publisher.
