# Supplementary material for: Vegetation History and Survival Patterns of the Earliest Village on the Qinghai–Tibetan Plateau
Source: Front Plant Sci. 2022 May 12;13:903192. doi: 10.3389/fpls.2022.903192 (PMC9134012; doi:10.3389/fpls.2022.903192)
Supplement: Supplementary file 1 [file Table_1.DOC]

**Supplementary Table 1.** Stratigraphic description and archaeological findings, section T1406E at Shalongka (SLK) site.

| Depth (cm) | Layer | Stratigraphy | Archaeological findings |
| --- | --- | --- | --- |
| 0–50 | 1–2 | Yellowish-brown silty | Historical period – modern accumulation |
| 50–78 | 3–4 | Grayish-brown silty | A very small number of Qijia culture pottery pieces in layer 4, representing secondary accumulation |
| 78–90 | 5 | Grayish-brown silty | Many pottery pieces of Qijia culture and a few of Machang type of Majiayao culture, and house sites, column caves and ash pits |
| 90–238 | 6–12 | Brownish-yellow and interbedded silty sand, occasional plant roots | Absent |
| 238–268 | 13 | Gray-black silty | Pottery pieces, pottery rings, stone rings and a few microliths of Miaodigou type of Yangshao culture |
| 268–292 | 14–16 | Dark-red sandy silt | Absent |
| 292–318 | 17–19 | Grayish-yellow silty | More than 400 microliths were unearthed, including stone anvils, microblade core, microblade, flakes and fragment, and 7 post holes arranged in a line at the base |
| 318–345 | 20–23 | Dark-red sandy silt | Absent |
| 345–368 | 24 | Milky yellow silt | A hearth, and a few flakes and fragments |
| 368–388 | 25–28 | Silty sand | Absent |
| 388–403 | 29 | Sandy silt | A few microblades and fragments |
| ＞403 | 30 | Brownish-gray sandy soil | Absent |
