# Supplementary material for: Vegetation History and Survival Patterns of the Earliest Village on the Qinghai–Tibetan Plateau
Source: Front Plant Sci. 2022 May 12;13:903192. doi: 10.3389/fpls.2022.903192 (PMC9134012; doi:10.3389/fpls.2022.903192)
Supplement: Supplementary file 2 [file Table_2.DOC]

**Supplementary Table 2.** AMS ^14^C dates from Shalongka (SLK) site.

| Lab number | Stratum | Dating material | ^14^C dating (a BP) | Calibrated age (2σ: cal a BP) | Reference |
| --- | --- | --- | --- | --- | --- |
| BA 161048 | Cultural layer 6 | Charcoal | 1940±25 | 1747 (2.6%) 1764 1783 (5.0%) 1813 1817 (92.4%) 1937 | This study |
| BA 161049 | Siltation layer 10 | Charcoal | 5390±45 | 6005 (17.5%) 6082 6110 (15.8%) 6152 6168 (66.7%) 6290 | This study |
| BA 161051 | Siltation layer 14 | Charcoal | 6605±30 | 7429 (72.5%) 7517 7533 (27.5%) 7567 | This study |
| BA 161052 | Cultural layer 17 | Charcoal | 6865±30 | 7618 (8.6%) 7646 7655 (91.4%) 7779 | This study |
| BA 161054 | Cultural layer 19 | Charcoal | 6760±30 | 7574 (100%) 7668 | This study |
| BA 161055 | Siltation layer 20 | Charcoal | 6875±30 | 7621 (100%) 7638 7657 (100%) 7789 | This study |
| BA 161056 | Cultural layer 24 | Charcoal | 6970±30 | 7700 (92.3%) 7864 7899 (7.7%) 7922 | This study |
| BA 161057 | Cultural layer 29 | Charcoal | 7640±30 | 8377 (89.7%) 8465 8470 (2.1%) 8483 8494 (8.2%) 8519 | This study |
| BA 161058 | Siltation layer 30 | Charcoal | 7645±30 | 8380 (89.6%) 8486 8491 (10.4%) 8519 | This study |
| LZU 16176 | Cultural layer 5b | Charcoal | 3650±30 | 3885 (72.6%) 4019 4025 (27.4%) 4085 | Wang et al., 2021a |
| LZU 16177 | Siltation layer 6 | Charcoal | 4955±35 | 5595 (99.3%) 5746 5833 (0.7%) 5838 | Wang et al., 2021a |
| LZU 16178 | Siltation layer 12a | Charcoal | 4415±25 | 4869 (93.4%) 5052 5190 (4.5%) 5217 5222 (1.0%) 5233 5248 (1.1%) 5260 | Wang et al., 2021a |
| LZU 16179 | Cultural layer 13 | Charcoal | 4200±25 | 4624 (1.9%) 4637 4642 (18.0%) 4679 4692 (51.3%) 4761 4796 (28.8%) 4841 | Wang et al., 2021a |
| LZU 16180 | Cultural layer 13 | Charcoal | 4345±30 | 4846 (98.4%) 4975 5014 (1.6%) 5023 | Wang et al., 2021a |
| LZU 16181 | Siltation layer 16 | Charcoal | 6955±25 | 7690 (98.6%) 7846 7904 (1.4%) 7913 | Wang et al., 2021a |
| LZU 16182 | Cultural layer 17 | Charcoal | 6895±30 | 7668 (99.0%) 7792 7818 (1.0%) 7823 | Wang et al., 2021a |
| LZU 16183 | Cultural layer 18 | Charcoal | 6950±25 | 7688 (99.9%) 7844 7909 (0.1%) 7910 | Wang et al., 2021a |
| LZU 16184 | Cultural layer 19 | Charcoal | 7060±30 | 7798 (2.2%) 7808 7834 (97.8%) 7961 | Wang et al., 2021a |
| LZU 16185 | Cultural layer 19 | Charcoal | 6655±30 | 7434 (3.2%) 7444 7470 (96.8%) 7578 | Wang et al., 2021a |
| LZU 16186 | Cultural layer 24a | Charcoal | 6530±30 | 7335 (15.0%) 7382 7420 (83.2%) 7507 7546 (1.8%) 7556 | Wang et al., 2021a |
| LZU 16187 | Cultural layer 24a | Charcoal | 6850±25 | 7615 (100%) 7740 | Wang et al., 2021a |
| LZU 16188 | Cultural layer 24b | Charcoal | 7130±30 | 7871 (12.7%) 7893 7928 (87.3%) 8013 | Wang et al., 2021a |
| LZU 16189 | Cultural layer 24b | Charcoal | 6620±30 | 7431 (14.0%) 7455 7460 (48.9%) 7522 7527 (37.2%) 7570 | Wang et al., 2021a |
| LZU 16190 | Siltation layer 26 | Charcoal | 6940±30 | 7682 (100%) 7841 | Wang et al., 2021a |
| LZU 16191 | Cultural layer 29 | Charcoal | 7675±30 | 8405 (100%) 8504 | Wang et al., 2021a |
| LZU 16192 | Cultural layer 29 | Charcoal | 7525±30 | 8210 (14.8%) 8259 8308 (85.5%) 8402 | Wang et al., 2021a |
| LUG 10128 | Cultural layer | Charcoal | 7535±58 | 8193 (25.4%) 8271 8277 (74.6%) 8418 | Dong et al., 2013 |
| Beta 297657 | Cultural layer | Charcoal | 7220±40 | 7951 (70.4%) 8054 8070 (29.6%) 8169 | Dong et al., 2013 |
| LUG 10185 | Cultural layer | Charcoal | 5121±88 | 5605 (1.4%) 5628 5651 (93.4%) 6014 6049 (0.6%) 6060 6077 (3.0%) 6114 6148 (1.6%) 6173 | Dong et al., 2013 |
| SLK 01 | Cultural layer | Charcoal | 2883±53 | 2868 (100%) 3167 | Li et al., 2014 |
| Beta 369356 | Cultural layer | Collagen from bones | 4480±40 | 4975 (8.2%) 5014 5024 (91.7%) 5299 | Ren, 2017 |
| LZU 151 | Cultural layer | Collagen from bones | 4460±20 | 4975 (14.7%) 5015 5024 (17.8%) 5083 5099 (12.3%) 5137 5165 (56.5%) 5281 | Ren, 2017 |
| Beta 292120 | Cultural layer | Charred millet seeds | 4340±41 | 4838 (93.4%) 4980 5006 (6.6%) 5036 | Dong et al., 2014 |
| Beta 292119 | Cultural layer | Charred millet seeds | 4340±40 | 4838 (94.1%) 4979 5007 (5.9%) 5035 | Chen et al., 2015 |
